# CRISPR/Cas9‐Mediated Base Editing of *SiGS1* Confers Glufosinate Resistance in Foxtail Millet (*Setaria italica*)

**DOI:** 10.1111/pbi.70440

**Published:** 2025-12-24

**Authors:** Jiayi Chen, Yangyang Zhang, Rui Zhao, Lingqian Zhang, Xuan Zhou, Xueting Kang, Yongchao Li, Shuqi Dong, Xiaorui Li, Lulu Gao, Guanghui Yang, Xiaoqian Chu, Xiangyang Yuan, Hongzhi Wang, Jia‐Gang Wang

**Affiliations:** ^1^ Special Orphan Crops Research Center of the Loess Plateau, Ministry of Agriculture and Rural Affairs College of Agriculture, Shanxi Agricultural University Taigu China; ^2^ Shanxi Hou Ji Laboratory College of Agriculture, Shanxi Agricultural University Taigu China

**Keywords:** CRISPR‐mediated base editing, foxtail millet, glufosinate resistance, SiGS1

Foxtail Millet [ *Setaria italica* (L.) P. Beauv.] is a drought‐tolerant, soil‐poor‐resistant crop whose yield is significantly limited by field weeds. Weed control remains a major constraint on its production and industrial development (Darmency et al. 2017). Developing non‐transgenic herbicide‐resistant germplasm offers a sustainable solution to mitigate production losses (Jin et al. 2022). Recent gene‐editing technologies represented by the CRISPR/Cas9 have enabled the specific enhancement of crop tolerance to herbicides through precision editing of crop herbicide target genes, with breakthrough progress already achieved in rice, maize, and other crops (Zhang et al. 2023). Therefore, it has become an urgent task to create herbicide‐resistant germplasm using this molecular method.

Glufosinate (phosphinothricin, PPT) is a widely used non‐selective, broad‐spectrum herbicide that is used globally to indirectly inhibit photosynthesis and culminate in the death of the plant by irreversibly inhibiting glutamine synthetase (GS), which leads to the high accumulation of ammonia (Takano et al. 2020). In this study, the identification and phylogenetic analysis of the GS gene in foxtail millet revealed the presence of five *SiGS* genes (Figure 1a). Complementary bioinformatic analyses—encompassing gene structure architecture, conserved motif identification, chromosomal localization, synteny assessment, and promoter *cis*‐element profiling—demonstrated that these five *SiGS* members reside across three chromosomes and exhibit lineage‐specific collinearity (Figure S1). Tissue‐specific expression profiling via *RT‐qPCR* further delineated functional divergence: *Si1G311400* displayed constitutive transcription across all examined organs (Figure 1b). This spatiotemporal regulation suggests subfunctionalization within the *SiGS* family, positioning *Si1G311400* (hereafter *SiGS1*) as the primary cytosolic GS isoform for subsequent functional investigation.

**FIGURE 1:**
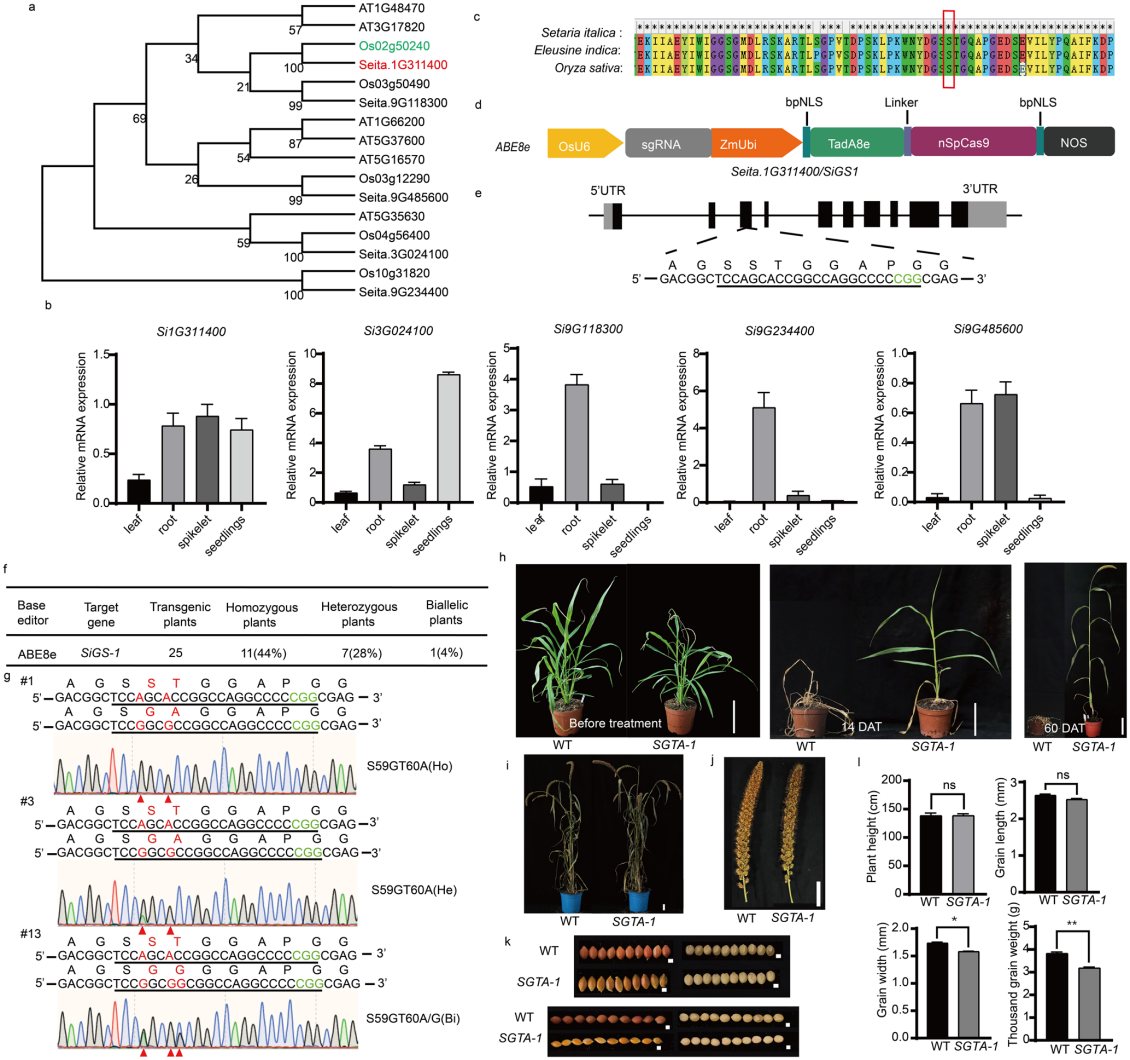
Engineering glufosinate‐resistant foxtail millet through base editing of *SiGS1*. (a) Phylogenetic reconstruction of glutamine synthetase (GS) proteins from foxtail millet ( *Setaria italica* ), rice ( *Oryza sativa* ), and *Arabidopsis thaliana* . *SiGS1* of foxtail millet is labelled in red, and the orthologous gene *OsGS1* of rice is labelled in green. (b) Tissue‐specific expression profiles of five *SiGS* genes quantified by *RT‐qPCR* in roots, leaves, spikelets, and seedlings. (c) Conserved domain alignment near Ser59 residue across foxtail millet (*SiGS1*), *E. indica* (*EiGS1*), and rice (*OsGS1*) proteins. The conserved S59G site has been highlighted with a red box. (d) Strategy for ABE8e‐mediated A•T to G•C conversion in *SiGS1* exon 3. (e) Schematic of sgRNA target design within *SiGS1*. (f) Editing efficiency distribution among 25 independent T_0_ transgenic lines. (g) Sanger sequencing chromatograms of edited alleles in lines #1, #3, and #13. PAM sequences (green), target sequences (underlined), nucleotide substitutions, and corresponding amino acid changes (red) are indicated. Edited alleles include S59G‐T60A (SGTA) and S59G‐T60A/G (SGTA/SGTG). (h) Phenotypic response of *SiGS1‐SGTA* and wild‐type (WT) plants 14 and 60 days after treatment with 1 g/L glufosinate. Untreated controls are shown. Scale bar = 10 cm. (i) Field phenotypic of wild‐type and *SiGS1‐SGTA* plants. Scale bar = 10 cm. (j‐k) Developmental characters and grain morphology of the panicle of wild‐type and *SiGS1‐SGTA* plants. The scale represents 5 cm and 1 mm. (l) Number of Plant height, thousand grain weight, grain length and width. Values shown as mean ± SD.

Building upon established evidence that concurrent overexpression of *OsGS1;1* and *OsGS2* confers glufosinate resistance in rice (James et al. 2018). To further verify the bioinformatic predictions, we transiently expressed *SiGS1* and tagged it with a GFP in *Nicotiana benthamiana* leaves. Confocal microscopy revealed exclusive cytoplasmic localization (Figure S2). Subsequently, we constructed overexpressing *SiGS1* (*SiGS1‐OE*) lines in Arabidopsis (Figure S3a). Then, we carried out a quantitative evaluation of glufosinate. The results after 2 weeks showed that *SiGS1‐OE* plants had limited tolerance to glufosinate at concentrations of 0.96 mg/L and 0.32 mg/L (Figure S3b). To definitively establish *SiGS1* as the functional glutamine synthetase isoform governing glufosinate sensitivity, we engineered knockout mutants in the elite foxtail millet cultivar Ci846 using CRISPR/Cas9‐mediated targeted mutagenesis. Molecular characterisation of T_0_ transformants confirmed five independent frameshift alleles (Figure S4a). Homozygous T_1_ generation (*SiGS1‐ko2*) was sensitive to glufosinate after 2 weeks of treatment (0.5 g/L, 1 g/L, and 2 g/L; Figure S4b).

Beyond the above two strategies, structural modification of glutamine synthetase represents an alternative approach to confer glufosinate resistance by reducing herbicide binding affinity (Zhang et al. 2022). Sequence alignment of GS1 orthologs revealed absolute conservation of Ser59 across angiosperms (Figure 1c), positioning this residue as an optimal target for precision editing in foxtail millet. We employed base editing‐mediated gene evolution (BEMGE) using the high‐efficiency adenine base editor ABE8e (Wang et al. 2022), which catalyzes A•T → G•C conversions coupled with optimized sgRNAs targeting *SiGS1* exon 3 (Figure 1d,e). Following *Agrobacterium*‐mediated transformation of foxtail millet cultivar Ci846, genomic DNA from 25 independently transgenic lines was analyzed by PCR amplification and Sanger sequencing, and 11 (44%) homozygous editors, 7 (28%) heterozygous editors, and 1 (4%) biallelic edited individual were identified (Figure 1f). Line #1 and line #3 carried the A_−17_–G, A_−14_–G conversions (S59G‐T60A, designated SGTA thereafter) in the targeting region. Line #13 carried the A_−17_–G, A_−14_–G and C_−13_–G/A conversions (S59G‐T60A/G, designated SGTA/SGTG thereafter) in the targeting region (Figure 1g). Subsequently, potential sgRNA‐dependent off‐target sites for *SiGS1‐SGTA* were predicted using the web tool CRISPR‐GE (http://skl.scau.edu.cn/). Two potential off‐target sites containing 2‐nt and 3‐nt mismatches relative to *SiGS1‐SGTA* were evaluated, and no off‐target events were detected across the 19 lines tested (Table S1).

To validate the herbicide resistance phenotype conferred by the *SGTA* allele, we exposed *SiGS1‐SGTA* and wild‐type plants to glufosinate challenge (1 g/L). Phenotypic assessment 14 days after application revealed a significant difference in response: the *SiGS1‐SGTA* plants remained viable, in contrast to the necrosis observed in wild‐type plants. Sixty days following glufosinate application, unlike the death of the wild type, the *SiGS1‐SGTA* plants grew normally (Figure 1h). The agronomic characteristics of glufosinate‐treated *SiGS1‐SGTA* plants were similar to those of wild‐type plants (Figure S7).

Prior to treatment, *SiGS1‐SGTA* plants exhibited substantially elevated basal GS activity compared to wild‐type counterparts. Following herbicide application, *SiGS1‐SGTA* plants maintained significantly higher enzymatic activity than wild‐type plants despite marked inhibition, indicating partial preservation of ammonium assimilation capacity. Concurrently, analysis of oxidative defence enzymes revealed comparable pre‐treatment levels of peroxidase (POD), superoxide dismutase (SOD), and catalase (CAT) activities across genotypes. Post‐application profiling demonstrated pronounced induction of these antioxidant systems exclusively in *SiGS1‐SGTA* plants, consistent with enhanced mitigation of herbicide‐induced oxidative stress (Figure S5a). Photosynthetic pigment quantification further corroborated this physiological resilience: chlorophyll a, chlorophyll b, carotenoids, and total chlorophyll displayed no genotypic differences before spraying. After glufosinate exposure, *SiGS1‐SGTA* plants retained significantly greater pigment levels than severely depleted wild‐type plants, confirming protection of photosynthetic apparatus integrity (Figure S5b). This aligns with base‐edited *GLR1* rice (Ren, Liu, et al. 2023). Collectively, our findings establish that CRISPR‐mediated S59G‐T60A substitution results in glufosinate‐resistant foxtail millet.

Research has shown that the growth rate of rice mutants lacking *OsGS1;1* is severely reduced (Tabuchi et al. 2005). Fortunately, *SiGS1‐SGTA* plants did not show significant growth inhibition under normal growth conditions (Figures S6 and 1i–l) we will use cytosine base editors (CBEs) and adenine base editors (ABEs) to generate additional glufosinate‐resistant alleles (e.g., *AVPS, +AF*, *D171N*, *H249Y*) as previously reported (Ren, Kuang, et al. 2023), and design a multi‐target base‐editing library covering the full‐length *SiGS* gene to screen for novel resistance loci. Ultimately, we will pursue multiple resistance alleles to create foxtail millet materials with enhanced glufosinate tolerance.

## Supporting information


**Appendix S1:** pbi70440‐sup‐0001‐AppendixS1.doc.

**Appendix S2:** pbi70440‐sup‐0002‐AppendixS2.docx.

## Data Availability

The data that support the findings of this study are available on request from the corresponding author. The data are not publicly available due to privacy or ethical restrictions.
