# Supplementary material for: CRISPR/Cas9‐Mediated Base Editing of SiGS1 Confers Glufosinate Resistance in Foxtail Millet (Setaria italica)
Source: Plant Biotechnol J. 2025 Dec 24;24(4):2592–4. doi: 10.1111/pbi.70440 (PMC13140573; doi:10.1111/pbi.70440)
Supplement: Supplementary file 1 — Appendix S1: pbi70440‐sup‐0001‐AppendixS1.doc. [file PBI-24-2592-s001.doc]

****Materials and Methods****

****1. Plant Materials and Growth Conditions****

The foxtail millet (Setaria italica) cultivar "**Ci846**" was used in this study as the wild-type (WT) control and for genetic transformation. Wild-type and *SiGS1*-edited plants were grown in a growth chamber under short-day conditions (10 h light at 28°C/14 h dark at 25°C) or in the field under natural conditions in Jinzhong, Shanxi, China.

***Nicotiana benthamiana* plants were grown in a greenhouse (16 h light / 8 h dark, 22 ± 2°C) for 4 weeks and used for transient expression assays.**

Arabidopsis thaliana ecotype ”**Col-0**”**** was used for overexpression assays. Seeds were surface-sterilized and grown on 1/2 MS medium for one week. In soil under long-day conditions (**16 h light / 8 h dark** at **22°C**).

****2. Identification and Bioinformatic Analysis of** SiGS **Gene Family****

****2.1 Identification of GS Genes****
The protein sequences of known glutamine synthetase (GS) genes from Arabidopsis thaliana and Oryza sativa were used as queries to perform BLASTP searches (E-value < 1e-10) against the Setaria italica v2.0 genome in the Phytozome v13 database to identify all putative SiGS genes.

****2.2 Phylogenetic Analysis****
A phylogenetic tree was constructed using the Neighbor-Joining (NJ) method in MEGA11 software with 1000 bootstrap replicates, based on the alignment of full-length GS protein sequences from foxtail millet, rice, and Arabidopsis.

****2.3 Gene Structure and Motif Analysis****
Gene structures (exon-intron structures) were visualized using the Gene Structure Display Server (GSDS 2.0). Conserved protein motifs were identified using the MEME suite (v5.5.0) with the following parameters: maximum number of motifs = 10; motif width = 6-50 amino acids. The results were integrated and visualized using TBtools software.

****2.4 Chromosomal Localization and Synteny Analysis****
The chromosomal locations of the SiGS genes were mapped using MapInspect software. Synteny analysis between foxtail millet and rice was performed using MCScanX and visualized with Circos.

****2.5 Promoter Analysis****
The genomic sequences spanning 2000 bp upstream of the translation start codon (ATG) of each SiGS gene were extracted from the Phytozome database. Putative cis-acting elements within these promoter regions were predicted using the PlantCARE database. The elements were categorized by function and visualized using TBtools.

****3.** RNA Extraction and *RT-qPCR* Analysis**

**3.1 Tissue collection**

Roots, leaves, spikelets, and seedlings of foxtail millet were harvested, frozen in liquid nitrogen, and stored at -80°C for subsequent RNA extraction.

**3.2 RNA extraction**

Total RNA was extracted from the frozen tissues using TRIzol reagent (Invitrogen, USA) according to the manufacturer's instructions. Genomic DNA contamination was removed by treatment with RNase-free DNase I (Thermo Fisher Scientific, USA). The quality and concentration of RNA were assessed using a Nanodrop spectrophotometer, and RNA integrity was verified by 1% agarose gel electrophoresis.

**3.3 *RT-qPCR***

First-strand cDNA was synthesized using the PrimeScript™ RT Reagent Kit (TaKaRa). qPCR was performed on a CFX96 Real-Time PCR System (Bio-Rad) using SYBR® Premix Ex Taq™ II (TaKaRa). Three biological replicates and three technical replicates were performed for each sample.

****4. Subcellular Localization of *SiGS1*****

The full-length coding sequence (CDS) of *SiGS1*(**without the stop codon**) was amplified and cloned into the **pCAMBIA3300-35S-EGFP** vector via homologous recombination (using a ClonExpress Ultra One Step Cloning Kit, Vazyme) to generate a C-terminal fusion with **Enhanced **Green Fluorescent Protein** (EGFP)** under the control of the **35S promoter**.

The recombinant plasmid was transformed into Agrobacterium tumefaciens strain GV3101 (pMP90). Positive colonies were grown, resuspended in infiltration buffer to an OD600 of ~0.8, and infiltrated into the abaxial side of leaves from 4-week-old N. benthamiana plants using a needleless syringe.

After **48-72 hours**, leaf discs were excised and observed under a **confocal laser scanning microscope (Leica TCS SP8)**. **EGFP** fluorescence was excited with a **488 nm laser**.

****5. Generation of***SiGS1***Overexpression Lines in***Arabidopsis***

The full-length *CDS* of *SiGS1* was cloned into the plant overexpression vector **pCAMBIA3300** to generate the **35S::SiGS1** construct. This construct was transformed into *Agrobacterium tumefaciens* strain **GV3101** and subsequently introduced into wild-type*Arabidopsis*(Col-0) plants using the standard **floral dip method**.

T_1_ seeds were screened on **½ MS plates containing 50 mg/L kanamycin**. Resistant seedlings were transplanted to soil, and T_2_ seeds were harvested from individual T_1_ plants. *RT-qPCR* was used to confirm *SiGS1* expression levels in T_1_ homozygous lines, with Col-0 as the control.

****6. CRISPR/Cas9-Mediated Knockout of***SiGS1***in Foxtail Millet****

A sgRNA of *SiGS1* was designed by CHOPCHOP. A specific sgRNA was designed and cloned into a millet-specific CRISPR/Cas9 binary vector (**pBUN421**).

The plasmid was sent to the company ( BioRun, Wuhan) for genetic transformation. Transgenic T_0_-generation plants were obtained, and genomic DNA was extracted from leaves of putative transgenic T_0_ plants using the **CTAB method**. The target region was PCR-amplified. The PCR products were purified and subjected to **Sanger sequencing**. The sequencing chromatograms were analyzed using software like SnapGene to identify and quantify insertion/deletion (indel) mutations.

****7. Base Editing of***SiGS1***Using ABE8e****

The adenine base editor ABE8e (TadA8e-nSpCas9) was used to mediate A•T→G•C conversions. A sgRNA targeting exon 3 of *SiGS1* (encompassing the Ser59 codon) was designed and cloned into the pYL-ABE8e vector (driven by the OsU6 promoter).

The construct was sent to Wuhan BioRun Company for genetic transformation, and the S59G locus of T_0_ generation plants was verified molecularly. For specific experimental methods, please refer to Section 6.

Genomic DNA was extracted from leaves of **25 independent T_0_ regenerated plants**. The target region was PCR-amplified and analyzed by **Sanger sequencing**. The editing efficiency was calculated as the **(Number of plants with desired edits / Total number of T_0_ plants analyzed) **× 100%****. Genotypes (homozygous, heterozygous, biallelic) were determined based on the sequencing chromatograms. Potential off-target sites were predicted using ****CRISPR-GE (****[**http://skl.scau.edu.cn/**](http://skl.scau.edu.cn/)****)**** and analyzed by sequencing.

****8. Herbicide Tolerance Assays****

**Phenotypes of *OE-SiGS1* Arabidopsis were assessed after two week of treatment with 6.76, 2.7, 0.32, or 0.96 mg/L glufosinate. *SiGS1-ko2* sensitivity was evaluated two weeks after treatment with 0.5, 1, or 2 g/L glufosinate. Phenotypes of *SiGS1-SGTA* were recorded at 14 days after treatment (DAT) with 1 g/L glufosinate. In the greenhouse, three-leaf-stage foxtail millet and four-week-old Arabidopsis seedlings were sprayed with glufosinate solution in a spray chamber at 0.3 MPa. Phenotypes were recorded 14 days after application, using wild-type non-transgenic plants as controls.**

****9. Physiological and Biochemical Assays****

Leaf samples from WT and *SiGS1-SGTA* plants were collected before (0 DAT) and 14 days after (14 DAT) glufosinate (1 g/L) treatment, frozen in liquid nitrogen, and ground to a fine powder.

Glutamine Synthetase (GS) Activity was measured using a Glutamine Synthetase Assay Kit (Boxbio, China) according to the manufacturer's protocol, based on the synthesis of γ-glutamylhydroxamate measurable at 540 nm. Activity was expressed as μmol γ-glutamylhydroxamate produced h⁻¹ mg⁻¹ protein (U mg⁻¹ prot).

Antioxidant Enzyme Activities were assayed as follows:

Superoxide Dismutase (SOD): Activity was assayed by measuring the inhibition of the photochemical reduction of nitroblue tetrazolium (NBT) at 560 nm. One unit (U) of SOD activity was defined as the amount of enzyme that caused 50% inhibition of NBT reduction per mg protein.

Peroxidase (POD): Activity was determined by monitoring the oxidation of guaiacol at 470 nm in a reaction mixture containing 50 mM phosphate buffer (pH 7.0), 20 mM H₂O₂, and 50 mM guaiacol. Activity was expressed as the change in absorbance per minute per mg protein (ΔA470 min⁻¹ mg⁻¹ prot).

Catalase (CAT): Activity was quantified by measuring the decomposition of H₂O₂ at 240 nm (extinction coefficient 39.4 mM⁻¹ cm⁻¹) in a reaction mixture containing 50 mM phosphate buffer (pH 7.0) and 15 mM H₂O₂. Activity was expressed as μmol H₂O₂ decomposed min⁻¹ mg⁻¹ protein (U mg⁻¹ prot).

Photosynthetic Pigments were extracted from ~0.1 g of fresh leaf tissue with 10 mL of 96% (v/v) ethanol in the dark at 4°C until colorless. Absorbances were measured at 665, 649, and 470 nm. The concentrations of chlorophyll a, chlorophyll b, total chlorophyll, and carotenoids were calculated using standard Lichtenthaler equations. Results were expressed as mg per gram fresh weight (mg g⁻¹ FW).

Statistical Analysis

All data are presented as mean ± standard deviation (SD). Statistical significance was determined by one-way analysis of variance (ANOVA) followed by Tukey's honestly significant difference (HSD) test using SPSS software (version 26.0). Differences were considered significant at p < 0.05.
