# Supplementary material for: CRISPR/Cas9‐Mediated Base Editing of SiGS1 Confers Glufosinate Resistance in Foxtail Millet (Setaria italica)
Source: Plant Biotechnol J. 2025 Dec 24;24(4):2592–4. doi: 10.1111/pbi.70440 (PMC13140573; doi:10.1111/pbi.70440)
Supplement: Supplementary file 2 — Appendix S2: pbi70440‐sup‐0002‐AppendixS2.docx. [file PBI-24-2592-s002.docx]

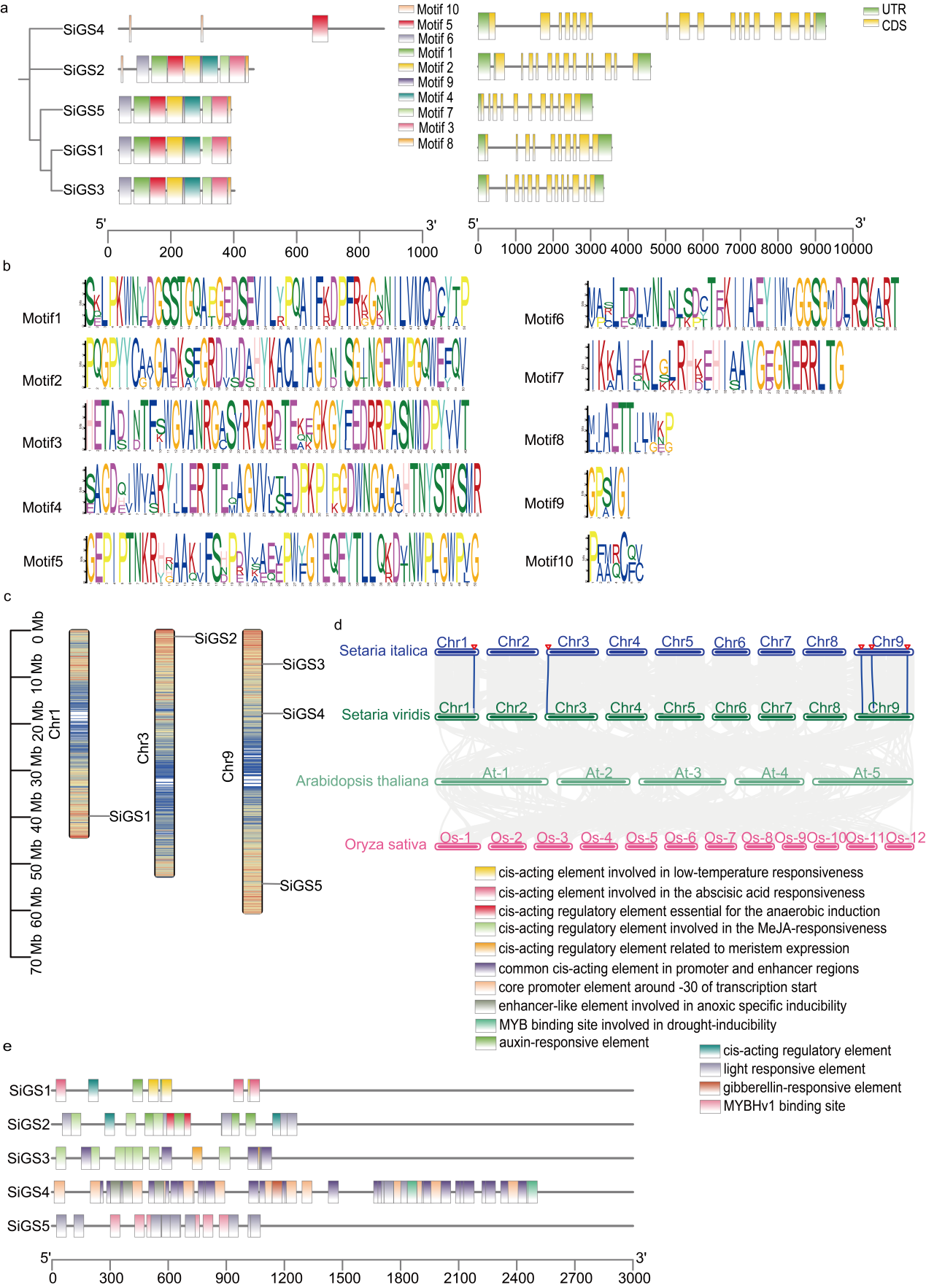


Figure S1 Genome-wide characterization analysis of the *SiGS* Gene Family in Foxtail Millet.

(a) Exon-intron structures of *SiGS* genes.

(b) Conserved protein motifs identified in five SiGS proteins.

(c) Chromosomal distribution of *SiGS* genes.

(d) Synteny analysis of GS genes among foxtail millet (*Setaria italica*), rice (*Oryza sativa*), *Arabidopsis thaliana* and *Setaria viridis.*

(e) cis-Element profiling in promoters of SiGS genes, highlighting functional associations with light response, phytohormones (MeJA, ABA, gibberellin), and abiotic stresses (drought, low temperature).


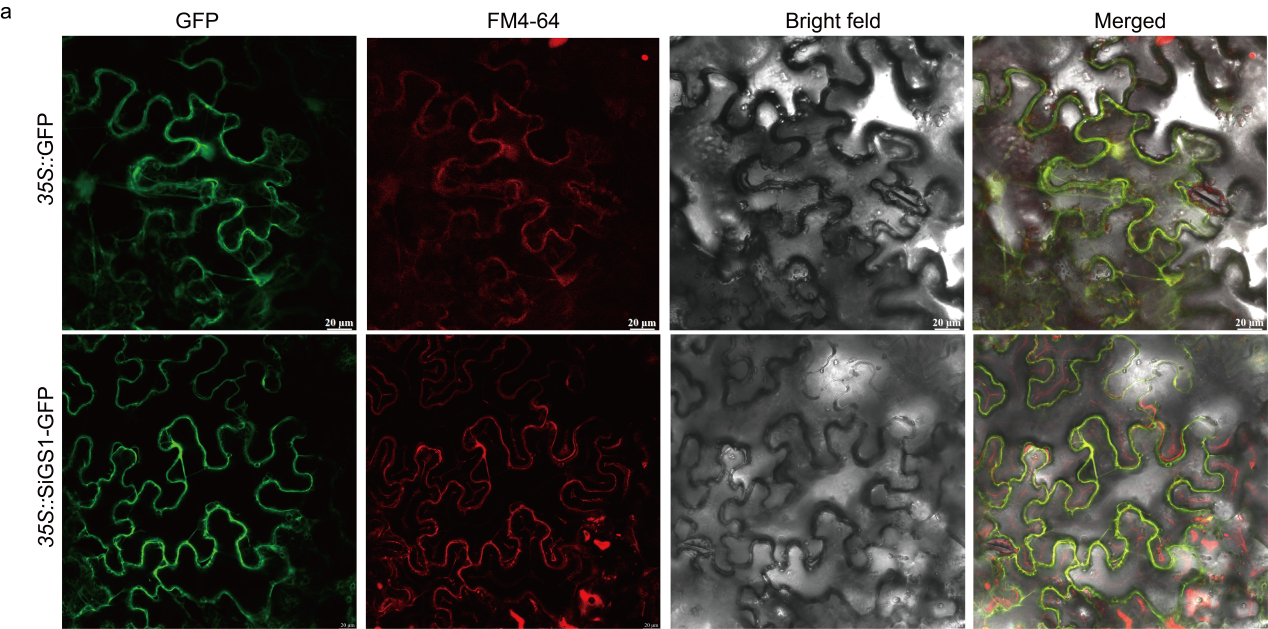


Figure S2 Subcellular localization of SiGS1 protein.

Transient expression of SiGS1-GFP fusion in *Nicotiana benthamiana* leaves. Confocal microscopy confirms exclusive cytoplasmic localization (GFP signal). Staining with FM4-64 was performed and merged images are shown. Scale bars = 20 µm


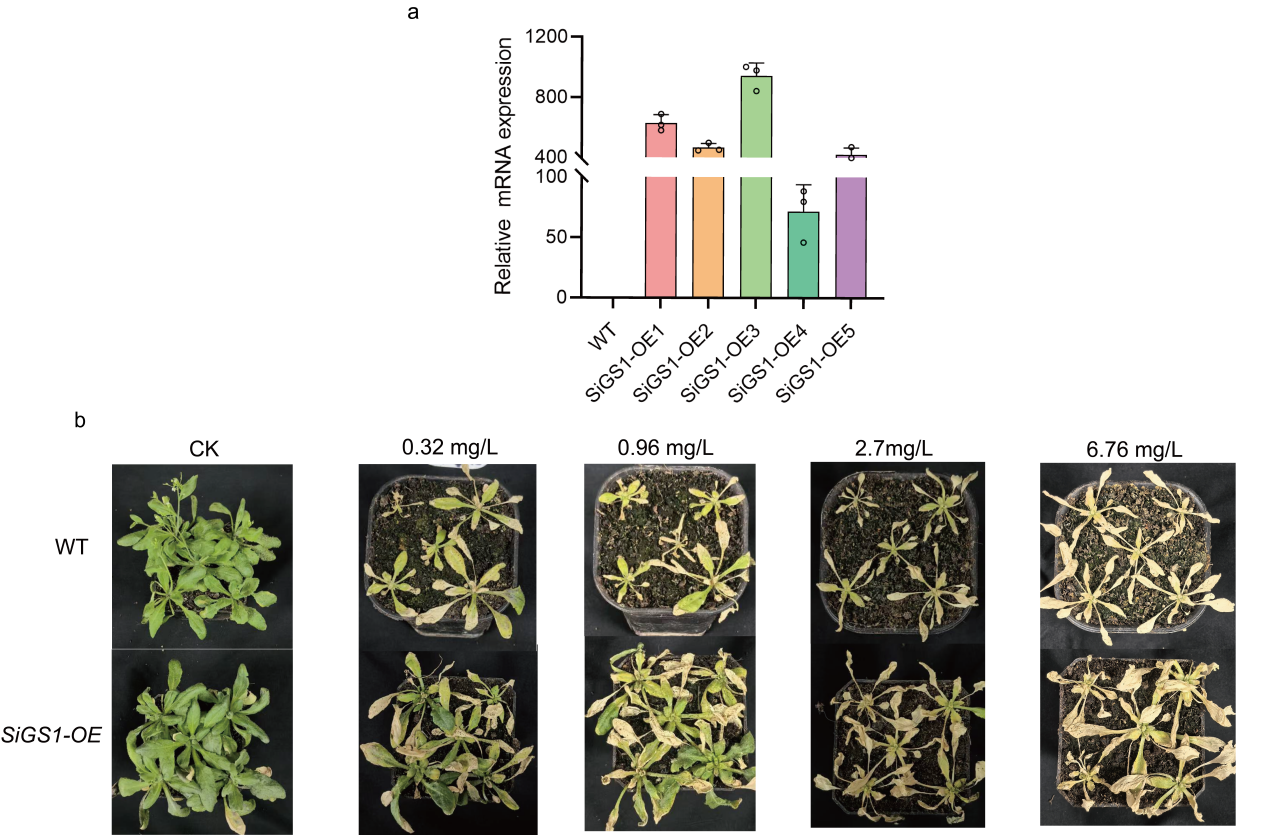


Figure S3 Functional validation of *SiGS1* overexpression in transgenic *Arabidopsis.*

1. Transgenic Arabidopsis plants overexpressing *SiGS1* under the control of the 35S promoter were generated, and five independent transgenic lines exhibited varying levels of *SiGS1* expression.

(b) Phenotypes of wild-type (WT) and *SiGS1-OE* transgenic *Arabidopsis* lines after 14-day exposure to glufosinate (0.96, 0.32, 2.7, and 6.76 mg/L). Untreated controls shown.


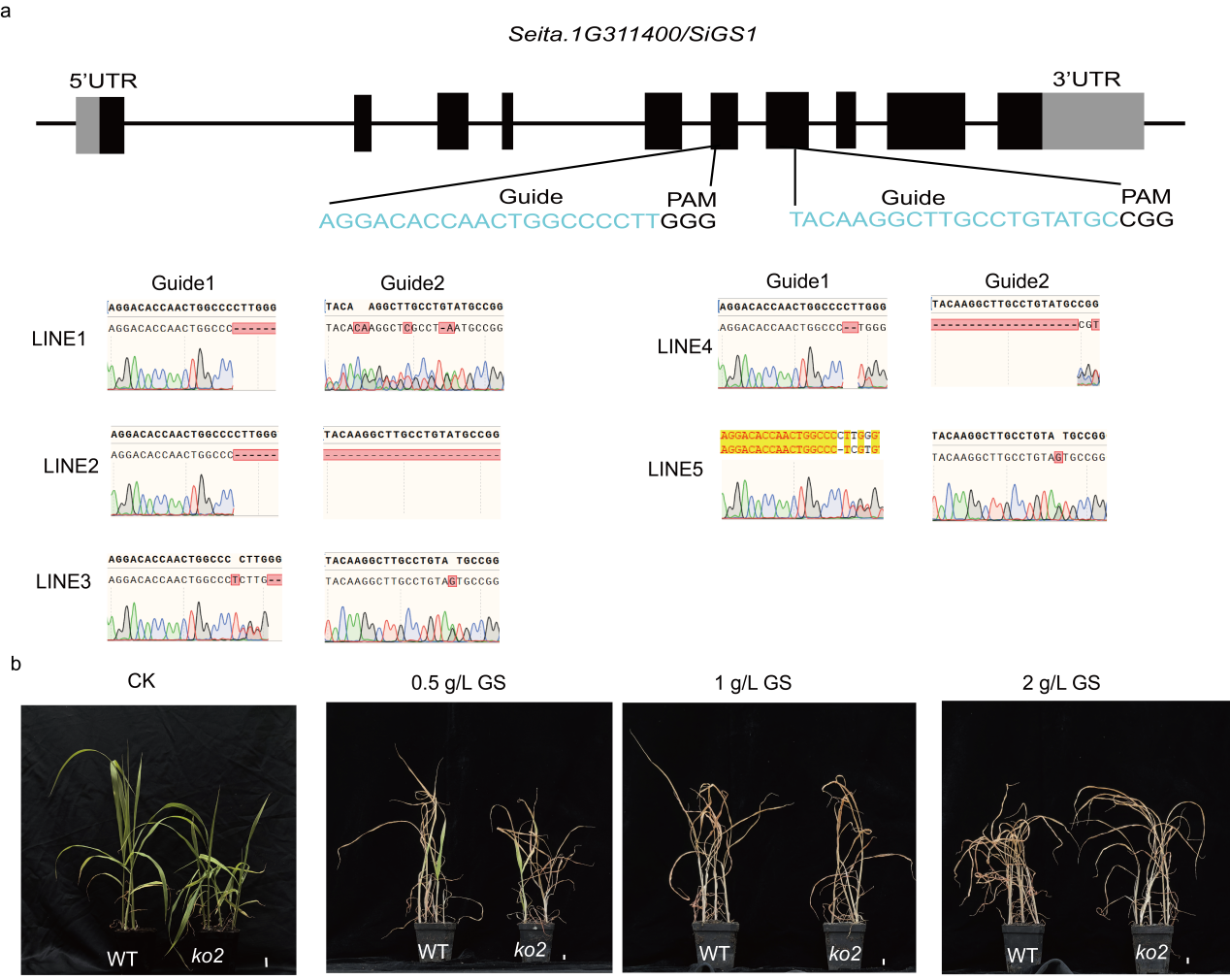


Figure S4 Generation and characterization of *SiGS1* knockout mutants.

(a) Target sites of sgRNAs in *SiGS1* and frameshift mutations in T₀ transgenic lines (#1, #2). PAM sequences shaded in black.

(b) Phenotypic response of wild-type (WT) and homozygous *SiGS1-ko2* T_1_ plants 14 days after treatment with glufosinate (0.5, 1, and 2 g/L). Untreated controls shown. Scale bar = 1 cm.


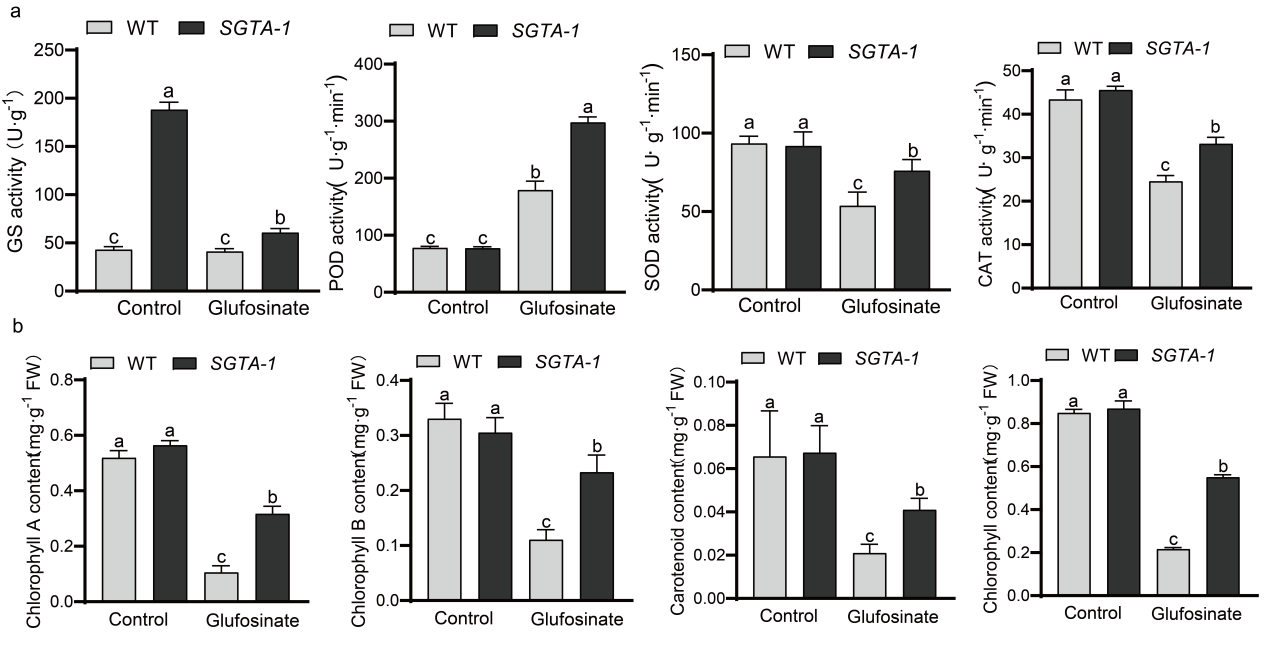
Figure S5 Physiological indices in wild-type and *SiGS1-SGTA* plants

(a) Glutamine synthetase (GS) activity and antioxidant enzyme activities (POD, CAT, SOD) in glufosinate-treated and untreated plants. Values shown as mean±SD.

(b) Photosynthetic pigment levels (chlorophyll A, chlorophyll B, carotenoids, total chlorophyll) in glufosinate-treated and control plants. Values shown as mean±SD.


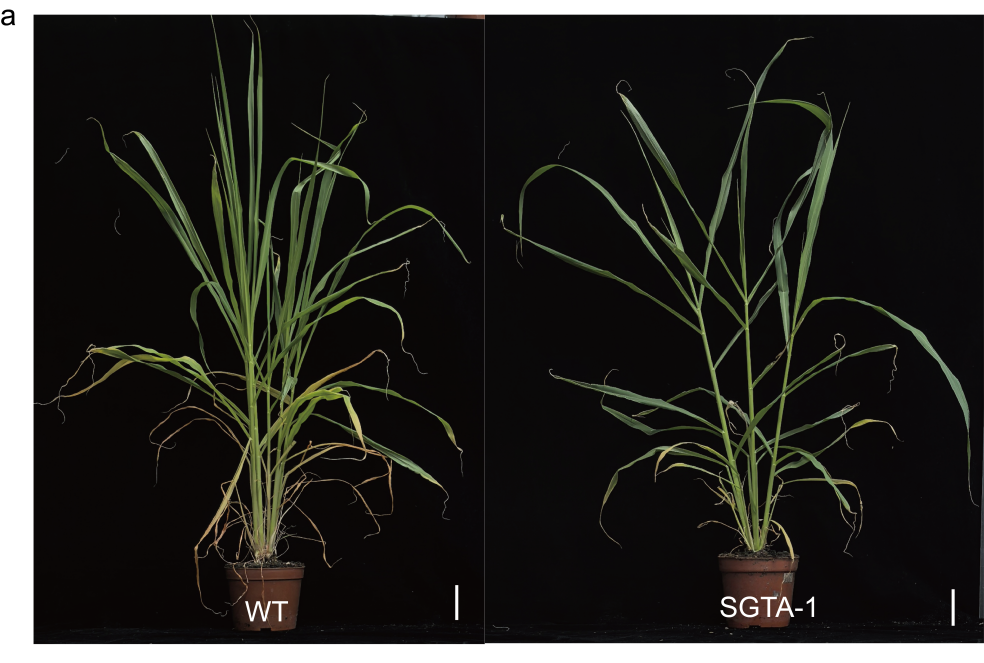


Figure S6 Growth phenotype of base-edited *SiGS1-SGTA* plants under control conditions.

*SiGS1-SGTA* (edited) and wild-type (WT) foxtail millet plants grown without glufosinate for 4 weeks. No significant growth inhibition was observed in edited lines. Scale bar = 10 cm.


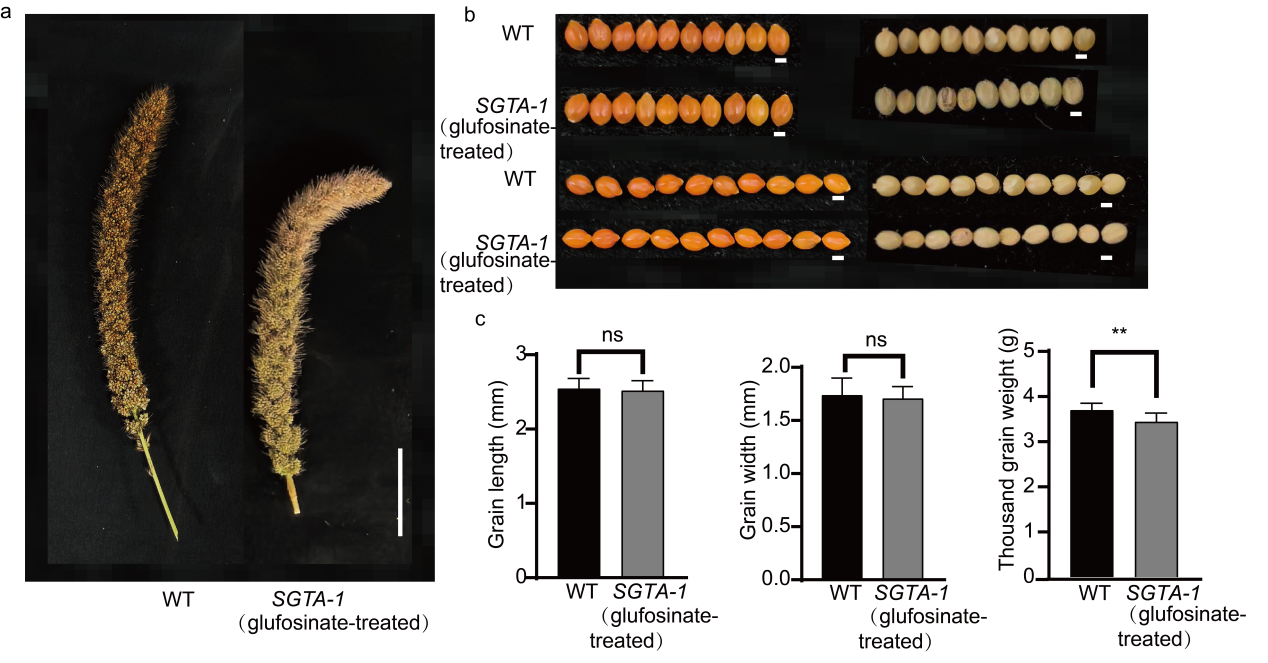
Figure S7 Phenotypic comparison of wild-type and glufosinate-treated *SiGS1-SGTA* plants.

(a-b) Developmental characters and grain morphology of the panicle of wild-type and glufosinate-treated *SiGS1-SGTA* plants. The scale represents 5 cm and 1 mm.

(c) Number of Thousand grain weight, Grain length and width. Values shown as mean ± SD.

| Target | Target site and potential off-target site | Putative off-target locus | Target region | No. of mismatching bases | No. of plants sequenced | No. of plants with mutations |
| --- | --- | --- | --- | --- | --- | --- |
| SiGS1-SGTA | On-target | *SiGS1* | TCCAGCACCGGCCAGGCCCCCGG | / | / | / |
|  | Off-target 1 | *Seita.9G485600* | TCCAGCACCGGGCAGGCTCCCGG | 2 | 19 | 0 |
|  | Off-target 2 | *Seita.8G226500* | TCGATCACCGGCCAGACCCCCGG | 3 | 19 | 0 |

Table S1 Potential off-target effects of gRNA used for *SiGS1-SGTA* mutation in the rice genome.

The PAM sequences and the mismatches to the target sequence in the potential off-target region are highlighted in green and red, respectively.

Primer sequences used in this study

| Name | Sequence (5'-3') |
| --- | --- |
| Primers for the overexpression vector constructed. | |
| 21-F | ACGGGGGACGAGCTCGGTACCATGGCCTGCCTCACCGACCT |
| 21-R | TGGCGCGCCGGGCCCTCTAGAGGGCTTCCAGATGATGGTGGTCT |
| Primers for constructing knockout vector. | |
| 22-F | AATAATGGTCTCAGGCGAGGACACCAACTGGCCCCTT |
| 22-F_0_ | GAGGACACCAACTGGCCCCTTGTTTTAGAGCTAGAAATAGC |
| 22-R_0_ | GCATACAGGCAAGCCTTGTACGCTTCTTGGTGCC |
| 22-R | ATTATTGGTCTCTAAACGCATACAGGCAAGCCTTGTA |
| Primers for identification of transgenic seedlings. (*SiGS1-ko*) | |
| 23-F | TGAACCATATGTTCCTGTGTTATACTGC |
| 23-R | AGACAACACAAAAAAGAGTTGGCATTG |
| Primers for the construction of the S59G vector. | |
| *SiGS1*-S59T60sg-F_1_ | TGTGTGCCAGCACCGGCCAGGCCCC |
| *SiGS1*-S59T60sg-R_1_ | AAACGGGGCCTGGCCGGTGCTGGCA |
| Primers for identification of transgenic seedlings. (*SiGS1-S59G*) | |
| *SiGS1*-F_2_ | ATCTCAGGAGCAAGGCTAGG |
| *SiGS1*-R_2_ | AAGGATGTTGTTGCCCTTCC |
| Primers for RT-qPCR quantification. | |
| 1G311400-F | CGAGAAGATCATCGCCGAGT |
| 1G311400-R | AGCTTGCTGGGATCAGTCAC |
| 3G024100-F | ATACGGAGAAGGAAATGAAA |
| 3G024100-R | CGAAAGTGCAACCCAC |
| 9G118300-F | GTTGGCGGTTCTGGGATG |
| 9G118300-R | TGAAGATGGCTTGAGGACGA |
| 9G234400-F | CGAAAGTGCAACCCAC |
| 9G234400-R | ATGCTAAGGTCTCCGTCT |
| 9G485600-R | GTTGGCGGTTCTGGGATG |
| 9G485600-F | GCTTGAGGACGAAGGATGAC |
| Primers for potential off-target effects | |
| GS1-OFF-F1 | ACCTCAGGAGCAAAGCAAGG |
| GS1-OFF-R1 | TTGTCACCCCTCCTGAATGG |
| GS1-OFF-F2 | AGTACTGGATCGGCAAGAAC |
| GS1-OFF-R2 | AAGCAAGCTAGGATGCATGT |
